# Long non-coding RNAs affecting cell metabolism in cancer

**DOI:** 10.1186/s13062-022-00341-x

**Published:** 2022-10-01

**Authors:** Massimiliano Agostini, Mara Mancini, Eleonora Candi

**Affiliations:** 1 Department Experimental Medicine, University of Rome “Tor Vergata”, TOR, Via Montpellier,1, 00133 Rome, Italy; 2 IDI-IRCCS, Via Monti di Creta 104, 00166 Rome, Italy

**Keywords:** Breast cancer, Long non-coding RNAs, Cell metabolism

## Abstract

Metabolic reprogramming is commonly recognized as one important hallmark of cancers. Cancer cells present significant alteration of glucose metabolism, oxidative phosphorylation, and lipid metabolism. Recent findings demonstrated that long non-coding RNAs control cancer development and progression by modulating cell metabolism. Here, we give an overview of breast cancer metabolic reprogramming and the role of long non-coding RNAs in driving cancer-specific metabolic alteration.

## General overview on long non-coding RNAs

LncRNAs are transcripts, longer than 200 nucleotides, that are not translated in proteins [1, 2]. They are classified in several classes based on a very broad definition, that includes enhancer RNA (eRNA), long intergenic transcripts (lincRNAs), transcripts overlapping other non-coding transcripts both in the sense and anti-sense orientation, transcripts overlapping portion of protein-coding transcript. LncRNAs exhibit specific tissue expression and functions, they have been extensively studied as key regulators of cell physiology and pathology [3], and as diagnostic and prognostic markers in different cancers [4, 5]. Despite the very large number of annotated lncRNAs, only a minority of them has been extensively studied at the molecular level, allowing the identification of their mechanism of action. In general, lncRNAs act to regulate gene expression, they can directly affect chromatin remodelling (recruitment of activator and/or repressors) or they can interact with intracellular molecules using different strategies. Indeed, they can act as scaffolds to modulate the activity of other RNAs and proteins, they can sequester miRNAs, RNAs, proteins, they can regulate mRNA stability and splicing, and they can interfere with translation of mRNAs into proteins [6–8]. Recently, accumulating evidences suggested that lncRNAs act at different levels in tumours initiation and transformation in several tumours including breast cancer [9–16], also they are involved in metastasis and epithelial-to-mesenchymal transition [17–19]. They are also strongly associated to the metabolic reprogramming required for breast tumours development.

## Breast cancers and metabolic reprogramming

Cancer cells reprogram their cellular metabolism to sustain cell proliferation by providing appropriate levels of energy in the form of ATP, biosynthetic capacity, and the maintenance of balanced redox status. These metabolic phenotypes are achieved by using glycolysis/ tricarboxylic acid cycle (TCA) intermediates for biosynthesis and NADPH production, increasing demand for nitrogen, requiring of an exogenous supply of glutamine and de novo biosynthesis of lipids [20]. Breast cancer is the leading cause of cancer–related death in women and about 90% of breast cancer deaths are related to the formation of distant metastasis [21]. Breast cancer is a heterogeneous disease that can be classified in several subtypes according to histopathological classes or to the molecular signature [22, 23]. Classically, breast cancer complies with the expression of hormone receptor for estrogen (ER+) and/or for progesterone (PR+) and for the expression of the human epidermal growth factor receptor (HER2) [24–27]. Therefore, breast cancer has been conventionally classified in five subtypes; luminal A (ER+ /PR+ /HER2−), luminal B, HER2 positive and triple negative (ER−/PR−/HER2−) [28–30]. This conventional classification of breast cancer has improved the diagnosis and the targeted therapy in patients, resulting in a better clinical outcome. However, we still need to uncover specific biomarkers that can assist in diagnosis, prognosis and proper treatment for each patient [31, 32]. We have recently shown the involvement of the p53 family [33–38] and in particular of p63 via the regulation of Sonic Hedgehog in the control of breast cancer proliferation [39–44], its transcriptional target ZNF750 [45, 46], as well as p73 [47]. These molecular mechanisms also showed the involvement of long noncoding RNA uc.63 [48]. Metabolomics research have made a great contribution in understanding specific metabolic pathways that are associated with cancer pathogenesis [49–55], and it is now largely accepted that metabolic alterations may be used as biomarkers. Thus, here we highlight the current knowledge on the main metabolic alterations that has been described in breast cancer.

### Glycolysis

Alteration of the glycolytic pathway in cancer cells (Warburg effect) has been the first metabolic reprogramming to be described [56].

Several experimental evidences in triple negative breast cancer (TNBC) cell line and patient samples have shown an enhanced glycolysis [57, 58]. In agreement, by characterizing glucose consumption, glutamine consumption, and glutamine dependence, it has been shown that ER+ and TNBC cell lines are highly glycolytic [57]. One possible explanation for the glycolytic phenotype of ER+ breast cancer cell lines is that ERα mediates the transcriptional activation of the hypoxia inducible factor-1α (HIF-1α) [59]. HIF-1α has been previously reported to induce a glycolytic signature of metabolic genes [60] and hence, ERα can indirectly activate glycolysis via the activation of HIF-1α. To further support this explanation, HIF-1α is highly expressed in ER+ tumours [61]. Moreover, it has been shown that ER+ cell lines are more glycolytic than ER- cell lines [62]. The increase of glycolysis has been also observed in human tumour samples. Indeed, high levels of glucose transporter 1 (GLUT1) have been observed in TNBC tumours [63]. GLUT1 may also enhance invasion by localizing to the invasive edge of in vivo tumour models [64]. However, more recently has been shown that TNBC cell lines displayed a significant metabolic heterogeneity, being for instance MDA-MB-468 less glycolytic than MDA-MB-231 [65]. Several determinants have been demonstrated to contribute to enhanced glycolysis. For instance, the key enzymes, including hexokinase (HK), phosphofructokinase (PFK) and pyruvate kinase (PK), that play a key role in controlling glycolytic rate have been found upregulated or more enzymatically active in breast cancer tissues [66, 67]. An additional level of regulation of the glycolytic pathway is mediated by the Pyruvate dehydrogenase kinase 1 (PDK1). PDK1 by phosphorylating the enzyme pyruvate dehydrogenase (PDH), results in the inactivation of the PDH enzyme complex that thereby inhibits the conversion of pyruvate to acetyl-coenzyme A, which is then oxidized in the mitochondria to produce energy [68]. Breast cancer stem cells (BCSC) are a small subpopulation of breast tumours, which play a critical role metastasis formation and resistance to treatment [69, 70]. PDK1 has been found highly expressed in BCSC and its expression is required for the metabolic reprogramming toward the glycolysis upon hypoxic conditions [71]. Although the canonical role of glycolysis is to provide ATP and NADH, it should be also mentioned that the glycolytic pathway plays also a key role in providing metabolites for the biosynthesis of fundamental building blocks such as, amino acid and nucleotides. Indeed, the intermediate 3-Phosphoglycerate could be diverted into the serine synthesis [49]. Although serine is a nonessential amino acid, several cancer cells rely on de novo synthesis of serine, which is required for the biosynthesis of lipids, protein, nucleotide, and amino acids [72]. It has been shown that enzymes controlling nucleotides biosynthesis are also responsible for TNBC de-differentiation [73]. The first step of this pathway is the oxidation of 3-Phosphoglycerate to 3-phosphohydroxypyruvate by the enzyme Phosphoglycerate dehydrogenase [74, 75]. Genomic amplification of the Phosphoglycerate dehydrogenase enzyme has been found in 6% of human ER-negative breast cancer [76, 77].

### Mitochondrial and OXPHOS

Most of the energy required by the cells is produced in the mitochondria via oxidative phosphorylation (OXPHOS), making mitochondria the powerhouse of the cells [78]. However, beyond producing energy, mitochondria are also involved in many processes including, generation of metabolites, biosynthetic metabolism, production of reactive oxygen species and regulation of cell death; and this is an essential need for cancer cells. Large body of evidence indicates that mitochondria have pleiotropic roles in the pathogenesis of cancer, depending upon genetic, environmental, and tissue-of-origin differences between tumours [79]. Breast cancer displays a significant inter-tumour metabolic heterogeneity [80]. Indeed, the luminal-like MCF-7 (ER+ and PR+) cell line shows a higher mitochondrial respiratory rate compared to the basal-like and highly metastatic MDA-MB-231 (ER− and PR−) cell line [81]. At molecular level, this phenotype was associated to the downregulation in MDA-MB-231 cells of Succinate Dehydrogenase Complex Iron Sulfur Subunit (SDHB), the core catalytic subunit of the mitochondrial heterotetrameric complex II, involved in both the citric acid cycle and electron transport chain. In addition, the expression of the complex I NDUFB8 subunit in MDA-MB-231 cells was lower when compared to MCF-7 cells suggesting that the switch from mitochondrial respiration to glycolysis is required for metastasis formation. This switch is required for cancer cells to evade the excess of ROS that are produced during the detachment from the extracellular matrix during the metastatic process [82, 83]. Therefore, by limiting mitochondrial oxidative metabolism, the glycolysis enables cancer cells to avoid excess ROS generation from mitochondrial respiration and thus gain survival advantage for metastasis. Consistently, the transcription factors HIF promotes metastasis formation by suppressing oxidative metabolism [84, 85]. However, invasive breast cancer cells exploit the transcription coactivator peroxisome proliferator-activated receptor gamma, coactivator 1 alpha (PGC-1α) to enhance oxidative phosphorylation, mitochondrial biogenesis and the oxygen consumption rate. In agreement, in human patients affected by invasive breast cancers has been observed a strong correlation between PGC-1α expression in invasive cancer cells and the formation of distant metastases [86]. In addition, breast cancer cells that metastasize to the lung and bone display an increase of OXPHOS metabolism compared to liver metastatic breast cancer cells, which are more glycolytic [87].

### Lipid metabolism

Lipids are a heterogonous group of biomolecules that have a pivotal role in many biological functions including synthesis of biological membranes, working as secondary messenger and as energy source [88]. Cancer cells exhibit increased capacity of producing lipids not only for enabling the formation of lipid bilayers but also changes its composition in order to counteract the oxidative damage of the phospholipids [89]. A comprehensive lipidomics analysis in human breast tissues display an increase in de novo fatty acid synthesis [90]. In particular, the membrane phospholipids, such as palmitate-containing phosphatidylcholines, were increased in tumors when compared with normal breast tissues. Interestingly, ER- breast cancer shows high levels of this lipids and this was associated with cancer progression and patient survival. HER2 is a marker of poor prognosis, which is overexpressed in about 30% of breast cancer [91]. Interestingly, pharmacological inhibition of fatty acid synthase (FAS) suppressed p185^*HER2*^ expression suggesting a possible molecular link between FAS and HER2 [92]. Lipid droplets are cytoplasmic organelles that play important roles in lipid metabolism. Recently, several studies have described an increase in intracellular lipid accumulation in different tumours and it has been shown that they are involved in all the steps of the cancer pathogenesis, including initiation, promotion and progression [93]. Accumulation of cholesteryl ester (CE), one of the main components of lipid droplets, was significantly higher in Her2+ and TNBC than in Luminal A tumours, indicating and association between CE content and poor clinical outcome [94]. In addition, lipid accumulation was higher in Her2+ breast cancer, further supporting the involvement of cytoplasmic lipid accumulation in breast cancer development [95–97].

To summarizes, breast cancer exhibits metabolic plasticity that allows cancer cells to choose the best metabolic program to sustain tumour progression. Indeed, depending of the genetic and epigenetic alterations and microenvironments conditions (i.e. hypoxia, metastatic site) breast cancer cells fulfil their bioenergetics and biosynthetic needs, choosing between glycolysis, OXPHOS and lipid metabolism. Interestingly, previous, and recent findings, indicate that long non-coding RNA (lncRNAs) affect all these metabolic pathways [57–101]. LncRNAs also modulate hormone sensitivity and resistance in breast cancers [102, 103], see Table 1. Therefore, there are strong evidences indicating that metabolism-associated lncRNAs could be explored as novel therapeutic targets for hormone-refractory breast cancers and breast cancer in general. In this review we provide an overview of the significative findings related to lncRNAs involvement in breast cancer metabolic reprogramming.

**Table 1:** Characteristics of breast cancer-related lncRNAs affecting metabolism

lncRNAs	Chr; length	Type (inter/intragenic)	Localization (nucleous-cytosol)	Maturation (SPLICING)	References
BCAR4	Chr16; 3363 bp	Intergenic	Nucleus		[121]
FGF13-AS1	ChrX; 849 bp	Intragenic			[122]
H19	Chr11; 2362 bp	Intergenic			[123]
lincRNA-p21	Chr6; 3000 bp	Intergenic	Cytosol		[124]
LINK-A	Chr1; 3331 bp	Intergenic	Cytosol		[106]
YIYA	Chr1; 1907 bp	Intergenic	Nucleus		[125]
MEG3	Chr14;	Intergenic	Nucleus	Spliced	[126]
PCGEM1	Chr2; 1603 bp	Intergenic	Nucleus	Spliced	[127]
UCA1	Chr19;	Intergenic	Nucleus	Spliced	[128]
SNHG5	Chr6; 524 bp	Intergenic	Nucleus	Spliced	[129]
FGD5-AS1	Chr3; 1812 bp	Intergenic		Spliced	[130]
GHET1	Chr7; 2917 bp	Intergenic		Spliced	[131]
HIFAL (HIF-AS1)	Chr14; 652 bp	Antisense RNA	Nucleus		[132]
MIR210HG	Chr11; 2303 bp	Antisense RNA		Spliced	[133]
MAFG-AS1	Chr17; 1914 bp	Intergenic		Spliced	[134]
LINC00346	Chr13; 6322 bp	Intergenic			[135]
NEAT1	Chr11; 3756 bp	Intergenic	Nucleus	Spliced	[136]
SRA	Chr17; 2275 bp	Intergenic			[137, 138]
MTORT1	ChrM; 572 bp		Mitochondria		[139]
GAS5	Chr1; 632 bp	Intergenic	Mitochondria	Spliced	[140]

## LncRNAs and glucose metabolism

Tumor cells preferentially use glycolysis to produce ATP even in aerobic conditions. For this reason, glycolysis-dependent cancer cells will be prone to undergo metabolic reprogramming as they possibly need to compensate for lower energy production efficiency by glycolysis due to high turnover of cell proliferation. Below, we will report examples of relevant lncRNAs involved in the glucose metabolism in breast cancer. A more exhaustive overwiew of lncRNA affecting glucose metabolism is shown in Table 2.

**Table 2:** LncRNAs involved in breast cancer metabolism and their mechanisms of action

lncRNAs	Expres-sion	Cell localization	Function	Target/pathways (interactors)	Mechanism of actions	References
*lncRNAs affecting GLUCOSE metabolism*						
BCAR4	Up	Nucleus	Oncogenic	Hippo pathway	Coordinates the Hedgehog signaling to enhance the transcription of glycolysis activators HK2 and PFKFB3	[99]
FGF13-AS1	Down	Cytoplasm	Tumor suppressor	IGF2BPs and Myc	Reduce the half-life of c-Myc (Myc) mRNA by binding IGF2BPs and disrupting the interaction between them	[141]
H19	Up	Cytoplasm	Oncogenic	Pyruvate dehydro genase kinase 1 (PDK1)	Acts as a competitive endogenous RNA for miRNA let-7 to release Hypoxia-inducible factor 1α, leading to an increase in PDK1 expression	[71]
lincRNA-p21	Up	Cytoplasm	Oncogenic	HIF-1a	Inhibits the degradation of HIF-1α; activates GLUT1 and LDHA; promotes Warburg effect	[142]
LINK-A	Up	Cytoplasm	Oncogenic	HIF-1a	Prevents the degradation of HIF-1α; recruites LRRK2 to phosphorylate HIF-1α, which promotes Warburg effect	[100]
YIYA	Up	Cytoplasm	Oncogenic	PFKFB3	Promotes catalysis of glucose 6-phosphate to fructose-2,6-bisphosphate/fructose-1,6-bisphosphate via CDK6 phosphorylation	[105]
MEG3	Down	Cytoplasm	Tumor suppressor	PI3K/Akt/miR-21, HK2	Inactivates the PI3K/Akt pathway functioning as a ceRNA of miR-21	[88]
UCA1	Up	Nucleus	Oncogenic	HK2	Directs cell metabolism towards aerobic glycolysis through AKT and STAT3 activation	[143]
SNHG5	Up	Cytoplasm	Oncogenic	miR-299	Promotes the glycolysis of breast cancer cell through regulating BACH1 via targeting miR‑299	[144]
FGD5-AS1	Up	Cytoplasm	Oncogenic	miR-195-5p	Mediates breast cancer glycolysis by upregulating NUAK2 via sponging miR‑195‑5p	[145]
GHET1	Up		Oncogenic	HIF-1a and LATS1	Stabilizes the activity of the Hippo/YAP signaling pathway	[146]
HIFAL	Up	Cytoplasm	Oncogenic	hnRNPF	Induce translocation of PKM2/PHD3 complex to enhance HIF-1α transactivation	[132]
MIR210HG	Up	Cytoplasm	Oncogenic	HIF-1a	Potentiates HIF-1a translation via directly binding to its mRNA 5’-UTR	[57]
MAFG-AS1	Up	Cytoplasm	Oncogenic	miR-3196	Through the axis miR-3196/TFAP2A, MAFG-AS1 activates the JAK2/STAT3 signaling pathway	[147]
LINC00346	Up	Cytoplasm	Oncogenic	miR-148a/b	Increases breast cancer cell glycolysis by down-regulating miR-148a/b and inducing GLUT1 expression	[148]
*lncRNAs affecting LIPID metabolism*						
NEAT1	Up	Cytoplasm	Oncogenic	miR-124	NEAT1 and STAT3 form a feedback loop via sponging miR-124 to promote BC progression	[118]
SRA	Up					[138]
LINK-A	Up		Oncogenic	AKT and PIP3	Facilitates AKT recruitment to PIP3 and enzymatic activation of AKT	[100]
*Mitochondrial lncRNA*						
MTORT1	Down	Mitochondria	Tumor suppressor	miR- 26a-5p	Serves as sponge of miR-26a-5p to up-regulate its target genes, CREB1 and STK4	[139]
GAS5	Down	Mitochondria	Tumor suppressor	MDH2 and SIRT3	Modulates mitochondrial tricarboxylic acid flux by disrupting metabolic enzyme tandem association of fumarate hydratase, malate dehydrogenase and citrate synthase	[101]

### LncRNA BCAR4

**Fig. 1:**
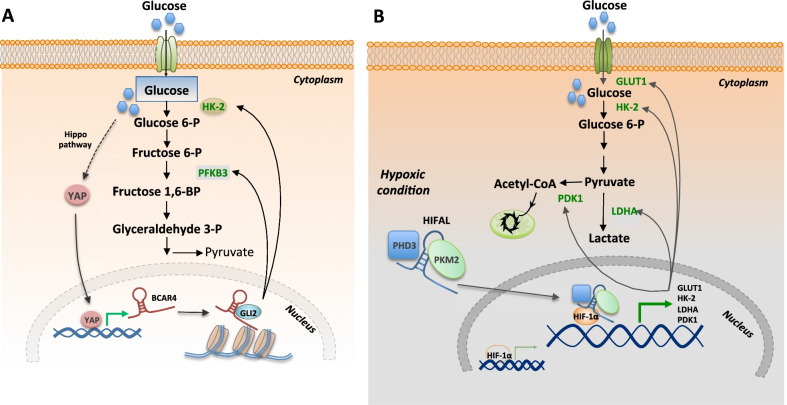
LncRNA regulates glycolysis in breast cancer cells. **A** YAP regulate the expression of the lncRNA BCAR4, which in turn by interacting with GLI2, promotes the transcription of two enzymes, HK2 and PFKFB3, resulting in the upregulation of the glycolytic pathway. **B** Under hypoxic conditions the LncRNA HIFAL is essential for maintaining HIF-1α transactivation and stimulates glycolysis in breast cancer cells by regulating the expression of glycolytic genes. HK-2: hexokinase 2; PFKB3: 6-Phosphofructo-2-Kinase/Fructose-2,6-Biphosphatase 3; GLUT1: Glucose Transporter 1; LDHA: Lactate Dehydrogenase A; PDK1: Pyruvate Dehydrogenase Kinase 1; GLI2: GLI Family Zinc Finger 2

BCAR4 (Breast cancer anti-oestrogen resistance 4) is a nuclear lncRNA with oncogenic function that regulates glycolysis. BCAR4 is a YAP target and it required for YAP-dependent regulation of glycolysis. Together with GLI2, BCAR4 promotes the transcription of two enzymes, HK2 and PFKFB3, positive effector of glycolysis in triple negative breast cancer cells. Interesting, if this regulatory axis BCAR4/GLI2-HK2/PFKFB3 is inhibited (using BCAR4 silencing or HK2 and PFKFB3 inhibitors) the YAP-dependent positive effect on glycolysis, and as consequence proliferation and tumorigenesis are suppressed [99] (Fig. 1A). In addition, previous studies have identified BCAR4 as an epigenetic regulator. BCAR4 associates with SNIP1 (Smad nuclear-interacting protein 1) and PPP1R10/PNUTS (serine/threonine-protein phosphatase 1 regulatory subunit 10 to promote the transcription of GLI2 (glioma associated oncogene homolog 2) modulating p300 histone acetyltransferase activity [104]. YAP-BCAR4 axis facilitates glycolysis and breast cancer metastasis. Clinically, BCAR4 and YAP expression are positively correlated in breast cancer and high expression of both BCAR4 and YAP is associated with poor survival of patients with breast cancer [99]. Besides BCAR4, other lncRNAs stimulates glycolysis in breast cancer cells enhancing the transcription of specific enzymes or supporting enzyme catalytic activity (Table 2), for example lncRNA YIYA that promote activation of the PFKFB3 enzyme [105].

### LncRNA LINK-A

The lncRNA LINK-A (Long Intergenic Non-coding RNA for Kinase Activation, also known as LINC01139) is a cytoplasmic lncRNA with oncogenic function, expressed in triple negative breast cancers. It is the first lncRNA described to interact with phospholipid [100]. LINK-A interacts with AKT and PIP3, to facilitate AKT activation. LINK-A-dependent AKT hyperactivation leads to tumorigenesis and resistance to AKT inhibitors. Clinically, LINK-A overexpression is observed in patients that develop resistance to AKT inhibitors. In addition, LINK-A binds and activates kinases such us breast tumour kinase (BRK) and leucine rich-repeat kinase 2 (LRRK2). HIF1a phosphorylation results in prevention of HIF1a degradation and enhance HIF1a transcriptional activity. These molecular events promote Warburg effects [106].

### LncRNA HIFAL

The lncRNA HIFAL (HIF-1α anti-sense lncRNA) is cytoplasmic lncRNA with oncogenic functions. LncRNA HIFAL is essential for maintaining HIF-1α triggered transcription under hypoxia condition and glycolysis in breast cancer cells.

The HIF-1α contributes to the Warburg observed in cancer cells, facilitating the switch from oxidative phosphorylation to glycolysis (4). Indeed, HIF-1 target genes include genes coding glycolytic receptors and enzymes, such us the glucose transporter GLUT1, the hexokinase II (HKII), the lactate dehydrogenase A (LDHA), and the pyruvate dehydrogenase kinase 1 (PDK1) [84, 107–109], necessary to switch the tumor cells from oxidative to anaerobic glycolysis in order to adapt to tumor hypoxic condition [110, 111] (Fig. 1B).

HIFAL is an important target of HIF-1 and can serve as a marker of HIF-1 mediated transactivation. HIFAL binds prolyl-hydroxylase 3 (PHD3) to pyruvate kinase 2 (PKM2) to stimulates PKM2/PHD3 complex migration into the nucleus, where interacting with HIF-1α facilitate its transcriptional activity. This is also relevant from a clinical point of view. Indeed, high HIFAL expression is associated with aggressive breast cancer phenotype and poor patient outcome. Furthermore, in vivo studies confirmed that HIFAL overexpression promotes tumor growth, while targeting/inhibiting both HIFAL and HIF-1α significantly reduces cancer growth [98].

## Mitochondrial and oxphos lncRNAs

Mitochondrial OXPHOS has been considered as a second line metabolic pathway in cancers, while aerobic glycolysis has been assumed as a major energy resource in cancer. Yet, recent evidence indicated that OXPHOS plays also an important role in cancer energy metabolism [112]. Recently, a big effort has been done to investigate cellular organelle-associated lncRNAs and among them of particular interest played the mitochondria-associated lncRNAs.

### LncRNA GAS5

**Fig. 2:**
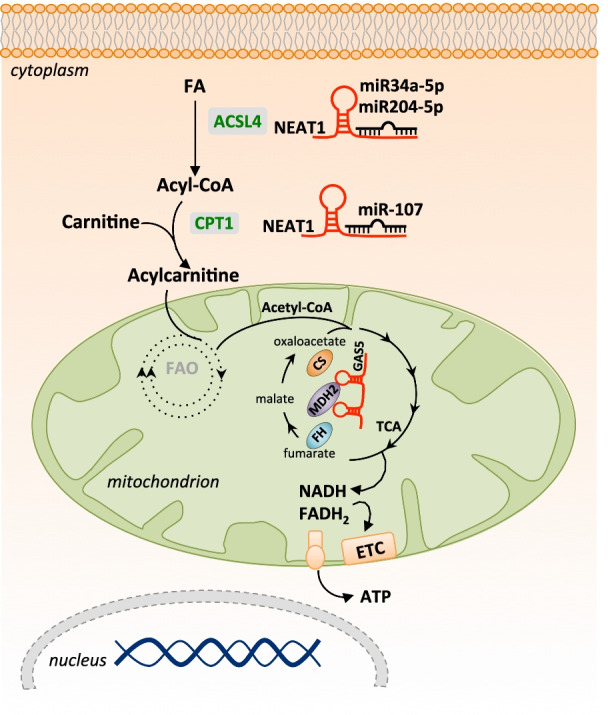
Regulation of mitochondrial activity and lipid metabolism by LncRNA in breast cancer. Energy–stress induces the expression of GAS5, which in turn inhibits the interaction between of FH, MDH2 and CS. This results in the reduction of TCA flux. The lncRNA NEAT stimulate the use of free fatty acids as energy source in breast cancer. NEAT1, sponging miR107, up regulates CPT1A expression in breast cancer cells. Competing with miR34a-5p and miR204-5p NEAT1 also controls the expression of ACSL4. TCA: Tricarboxylic acid; FH: Fumarate hydratase; CS: Citrate synthase; MDH2: Malate dehydrogenase; FAO: Fatty acid oxidation; ACSL4: Acyl-CoA Synthetase Long Chain Family Member 4; CPT1: Carnitine Palmitoyltransferase 1

GAS5 is a mitochondria-associated lncRNA very sensitive to cellular glucose levels. Previous studies have shown that it acts as DNA decoy for glucocorticoid receptor upon growth factor treatments. It also regulated insulin receptor transcription and possibly could work as sponge for miRNAs [32–114]. GAS5 is found associated to mitochondria. GAS5 reduce the TCA flux. Mechanistically, GAS5 block the association of fumarate hydratase (FH), MDH2 and citrate synthase (CS), by decreasing the MDH2 acetylation (Fig. 2). Clinically, datasets showing high expression of GAS5 with low TCA flux in breast cancer patients is associated with positive clinical outcomes [101]. These results, identifying a lncRNA mitochondria-associated, suggest that organelle-associated lncRNAs might have an important role in controlling cancer development. Furthermore, it gives evidence for a paradigm shift in the current understanding of cancer metabolism, giving importance also to the TCA cycle.

## LncRNAs involved in lipid metabolism

The high-energy demand required for cancer cell proliferation also takes advantage of the lipid utilization. Usually, cancer cells prefer to synthetize lipids using de novo pathways. As matter of facts, usually the expression of upregulated key lipogenic enzymes is up regulated in tumours [84, 92–115]. Interestingly, recent evidences indicated that lncRNAs not only directly interact with lipid (see LINK-A) but also play a role in potentiating lipid biosynthesis using different mechanisms.

### LncRNA NEAT1

The lncRNA NEAT1 (Nuclear Enriched Abundant Transcript 1) has been found deregulated in several human cancers including breast cancer. In particular, in breast cancer NEAT1 stimulates the use of free fatty acids as energy source. Indeed, in MDA-MB-231 (TNBC) and MCF7 (ER+/PR+) cancer cell lines NEAT1 by sponging miR-107, upregulates CPT1A expression [116]. CPT1A is a key enzyme for the synthesis of acylcarnitines, which are then transported into the mitochondria and its acyl groups are metabolized in the TCA cycle. In addition, competing with other miRNAs (miR34a-5p, miR204-5p) NEAT1 also controls the expression of ACSL4, essential for ER-negative breast cancer progression [117]; while competing with miR124 it form a positive loop including STAT3 to promote breast cancer progression [118] (Fig. 2). Interestingly, recent studies demonstrate that NEAT1 by sequestering the mRNAs encoding for mitochondria proteins in paraspeckles, also controls mitochondria dynamic and functions [119, 120].

## Conclusions

Here, we summarized the functions of cancer metabolism-related lncRNAs, focusing on breast cancer (Tables 1 and 2). LncRNAs involvement in cancer development has opened interesting research area from basic science to clinical research. Several studies support the use of lncRNAs as diagnostic, prognostic and/or predictive biomarkers. Understanding the role of lncRNAs in metabolic reprogramming may be instrumental for their use as monitoring tools and therapeutic targets, leading to improved personalized precision breast cancer medicine.

## Data Availability

Available upon requests.
